# Relationship between *Helicobacter pylori* infection and remnant cholesterol: the mediating role of insulin resistance and inflammation

**DOI:** 10.3389/fcimb.2025.1684556

**Published:** 2025-12-19

**Authors:** Yi Chen, Lingyan Shen, Ningning You, Jinshun Zhang

**Affiliations:** 1 Department of Gastroenterology, Taizhou Hospital of Zhejiang Province affiliated to Wenzhou Medical University, Taizhou, China; 2 Home ward, Taizhou Hospital of Zhejiang Province affiliated to Wenzhou Medical University, Taizhou, China; 3 Health Management Center, Taizhou Hospital of Zhejiang Province affiliated to Wenzhou Medical University, Taizhou, China; 4 Taizhou Hospital of Zhejiang Province, Shaoxing University, Taizhou, China

**Keywords:** *Helicobacter pylori*, inflammation, insulin resistance, mediation analysis, remnant cholesterol

## Abstract

**Background:**

*Helicobacter pylori* (*H. pylori*) represents a widespread chronic bacterial infection that has garnered increasing attention in recent years due to its extra-gastric effects. Remnant cholesterol (RC) is recognized as a non-traditional lipid marker and is a significant predictor of residual risk in atherosclerotic cardiovascular disease. This investigation aimed to analyze the correlation between *H. pylori* infection and RC levels, as well as to reveal the underlying mechanisms.

**Methods:**

The study population comprised individuals undergoing routine health examinations at the health examination center of Taizhou Hospital. All participants were subjected to urea breath tests, blood tests, and anthropometric measurements. The triglyceride-glucose (TyG) index was utilized to assess insulin resistance (IR) levels, while the erythrocyte sedimentation rate (ESR) was utilized as an indicator of chronic inflammation levels within the population. To assess the relationship between *H. pylori* infection and RC, a multiple linear regression analysis was carried out, also investigating the mediating roles of the TyG index and ESR.

**Results:**

Multiple linear regression analysis demonstrated a significant association between *H. pylori* infection and RC levels, with this relationship being stable across diverse populations. Mediation analysis further revealed that the TyG index and ESR significantly mediate the relationship between *H. pylori* and RC levels. Moreover, longitudinal analysis demonstrated that persistent *H. pylori* infection results in a marked increase in RC levels.

**Conclusion:**

Our research identified an association between *H. pylori* infection and elevated RC levels, with IR and inflammation acting as mediating factors in this relationship.

## Introduction

*Helicobacter pylori* (*H. pylori*) is a spiral-shaped, Gram-negative, microaerophilic bacterium characterized by multiple flagella, which predominantly colonizes the gastric mucosa in humans (Gu, 2017). It is one of the most common chronic bacterial infections worldwide (Engelsberger et al., 2024). Epidemiological data indicate that the global prevalence of *H. pylori* infection surpasses 50%, with even higher incidence rates reported in developing nations where sanitation is inadequate (Gravina et al., 2018). The World Health Organization has classified *H. pylori* as a Group 1 carcinogen, and it is definitively associated with various gastrointestinal diseases (Elshenawi et al., 2023). Recent research has increasingly highlighted that the pathological impact of *H. pylori* infection extends beyond localized gastrointestinal disturbances. *H. pylori* may induce systemic low-grade inflammatory responses, disrupt host immune regulatory functions, and impair metabolic signaling pathways, potentially impacting multiple distant organ systems (Bravo et al., 2018; He et al., 2022). In particular, its associations with cardiovascular and metabolic conditions, including atherosclerosis, non-alcoholic fatty liver disease, diabetes and lipid metabolism disorders, have attracted growing scholarly attention (Tsay and Hsu, 2018; Wang et al., 2020; Xie et al., 2023). These findings indicate that *H. pylori* is not solely a pathogenic element for gastric diseases but may also contribute to chronic systemic inflammation, posing a significant threat to the host’s metabolic homeostasis. However, the underlying mechanisms remain complex and necessitate further investigation.

Remnant cholesterol (RC) comprises the portion of cholesterol transported by intermediate-density lipoprotein cholesterol, very low-density lipoprotein (VLDL) cholesterol, and chylomicron remnants (Burnett et al., 2020). This cholesterol is not covered by the traditionally measured high-density lipoprotein (HDL) cholesterol and low-density lipoprotein (LDL) cholesterol, and it primarily resides in triglyceride-rich lipoprotein particles (Burnett et al., 2020; Wadström et al., 2023b). Recent researches have demonstrated that RC plays an independent and significant pathological role in the formation of atherosclerotic plaques and the incidence of cardiovascular events (Castañer et al., 2020; Xu et al., 2024; Li et al., 2025). Although LDL has traditionally been viewed as the primary atherogenic lipid factor, numerous clinical investigations have demonstrated that elevated RC levels are strongly correlated with residual risk for atherosclerotic cardiovascular disease, even when patients’ LDL levels are within target or optimal ranges (Sandesara et al., 2019; Heo and Jo, 2023).

Researches have indicated that individuals infected with *H. pylori* frequently exhibit dyslipidemia, potentially linked to mechanisms such as chronic low-grade inflammation, dysbiosis, and insulin resistance (IR) (Kim et al., 2016; Zhao et al., 2019; Martin-Nuñez et al., 2021; Qiu et al., 2025). The erythrocyte sedimentation rate (ESR) is a well-established nonspecific inflammatory marker that remains extensively used for monitoring chronic systemic inflammation due to its high sensitivity, low cost, and technical simplicity, making it effective in reflecting the body’s long-term inflammatory state (Erikssen et al., 2000; Corsonello et al., 2011). Similarly, the triglyceride-glucose (TyG) index, derived from fasting blood glucose (FBG) and triglyceride (TG) levels, has been proposed and widely adopted for the indirect assessment of IR (Lim et al., 2019; Tahapary et al., 2022). It offers significant advantages, including ease of use, low cost, good stability, and robust predictive capability, particularly demonstrating wide applicability in epidemiological studies and preliminary clinical screenings (Tao et al., 2022). While existing literature indicates a possible link between *H. pylori* and dyslipidemia, there remains a paucity of systematic research specifically examining the relationship between *H. pylori* and RC levels. Consequently, this study seeks to assess the potential association between *H. pylori* infection and RC levels utilizing a large population dataset, in addition to exploring the underlying mechanisms involved.

## Methods

### Study participants

This study encompassed individuals who participated in routine health examinations at the Taizhou Hospital Health Management Center between June 2017 and March 2024. We excluded individuals with incomplete clinical data, a history of malignancies, a history of gastrointestinal surgeries, severe cardiovascular or cerebrovascular conditions, those currently undergoing lipid-lowering medication treatment, and individuals experiencing acute infections. A total of 76,565 individuals participated in the study. Additionally, some individuals underwent multiple health examinations. Based on the status of *H. pylori* infection during the initial and final examinations (with a follow-up period exceeding one year), the participants were categorized into three groups: persistent infection (positive at both time points), persistent negative (negative at both assessments), and eradicated infection (transition from positive to negative), resulting in 12,654 individuals for further analysis. In this study, *H. pylori* was treated using the standard quadruple therapy regimen, which includes a proton pump inhibitor, a bismuth compound, and two antibiotics.

### Clinical index collection

Demographic information and lifestyle data of the participants, encompassing age, sex, smoking and alcohol consumption history, as well as medical history, were collected by trained nurses from the Health Management Center using a structured questionnaire. Subsequently, height, weight, seated systolic blood pressure (SBP) and diastolic blood pressure (DBP) were measured according to standardized protocols. All participants provided fasting venous blood samples after fasting for a minimum of eight hours, which were promptly sent to the laboratory for analysis. The laboratory tests included creatinine (Cr), TG, HDL, LDL, total cholesterol (TC), FBG, glycated hemoglobin (HbA1c), and ESR. RC was calculated using the formula: RC = TC − HDL − LDL. Body mass index (BMI) was defined as weight (kg) divided by height (m) squared (Huxley et al., 2010). The TyG index was calculated using the formula: ln [TG (mg/dL) × FBG (mg/dL)/2] (Simental-Mendía et al., 2008).

### Detection of *H. pylori*

Following an overnight fast, all participants underwent either the ^13^C or ^14^C breath test. The protocol for the ^13^C breath test involves the participant initially exhaling into a specialized collection bag after normal inhalation. Subsequently, the participant ingests a ^13^C-labeled urea capsule with water, ensuring that the capsule remains intact. After a 30-minute interval, the participant exhales into a second collection bag to complete the procedure, and the samples are then dispatched for analysis. In the case of the ^14^C breath test, participants directly ingest a capsule containing ^14^C-labeled urea with water. After remaining seated quietly for 15 minutes, they exhale slowly into a designated breath card. The samples are then forwarded for analysis.

### Statistical analysis

Categorical variables were analyzed using chi-square tests and reported as frequencies and percentages. Continuous variables were evaluated using independent t-tests or Mann-Whitney tests to assess differences between groups. The relationships between variables were assessed using Spearman correlation tests. To investigate the association between *H. pylori* and RC, multivariable linear regression analysis was conducted after adjusting for potential confounders. A generalized additive model was employed to examine the association between the TyG index and RC. Furthermore, a mediation analysis was performed to explore the mediating effects of the TyG index and ESR on the relationship between *H. pylori* and RC. All statistical analyses were carried out using R software (version 4.4.1), with statistical significance determined at a threshold of P < 0.05.

## Results

### Study participants

**Table 1** provides a comprehensive overview of the baseline characteristics of all participants. The cohort within the *H. pylori* group demonstrated a higher prevalence of males, smokers, and individuals who consumed alcohol. Furthermore, this group was characterized by an older age demographic and exhibited elevated levels of Cr, RC, SBP, DBP, HbA1c, FBG, TyG index, ESR, and BMI. Conversely, this group presented with reduced levels of TC, HDL, and LDL.

**Table 1:** Baseline characteristics of the study population.

Variables	*H. pylori* negative (n=52446)	*H. pylori* positive (n=24119)	P-value
Sex (n, %)			<0.001
Female	21093 (40.2)	9081 (37.7)	
Male	31353 (59.8)	15038 (62.3)	
Smoke (n, %)			0.001
No	45210 (86.2)	20567 (85.3)	
Yes	7236 (13.8)	3552 (14.7)	
Drink (n, %)			0.054
No	46559 (88.8)	21525 (89.2)	
Yes	5887 (11.2)	2594 (10.8)	
Age (years)	49.08 ± 12.55	49.80 ± 12.47	<0.001
Creatinine (μmol/L)	70.26 ± 19.04	71.04 ± 19.32	<0.001
Total cholesterol (mmol/L)	5.20 ± 1.03	5.16 ± 1.02	<0.001
High density lipoprotein (mmol/L)	1.43 ± 0.34	1.40 ± 0.32	<0.001
Low density lipoprotein (mmol/L)	2.86 ± 0.76	2.82 ± 0.76	<0.001
Triglyceride (mmol/L)	1.39 (0.96-2.08)	1.45 (0.98-2.22)	<0.001
Remnant cholesterol (mmol/L)	0.83 (0.64-1.08)	0.86 (0.66-1.09)	<0.001
Diastolic blood pressure (mmHg)	76.33 ± 11.83	76.91 ± 12.11	<0.001
Systolic blood pressure (mmHg)	127.17 ± 17.86	128.34 ± 18.85	<0.001
Fasting blood glucose (mmol/L)	5.43 ± 1.40	5.54 ± 1.65	<0.001
Glycated hemoglobin A1c (%)	5.87 ± 0.90	5.94 ± 1.01	<0.001
TyG index	8.72 ± 0.66	8.78 ± 0.70	<0.001
Erythrocyte sedimentation rate (mm/h)	7 (3–13)	7 (4–14)	<0.001
Body mass index (kg/m^2^)	24.38 ± 3.30	24.65 ± 3.32	<0.001

### Multiple regression analysis

To examine the impact of *H. pylori* on RC, a multiple linear regression analysis was performed, incorporating variables such as smoking, drinking, TG, Cr, SBP, FBG, ESR, and BMI, alongside sex and age. The findings, presented in **Table 2**, indicate that *H. pylori* remains a significant risk factor for RC. The correlations among these factors are illustrated in **Supplementary Figure S1**.

**Table 2:** Multiple linear regression analysis.

	B (95%CI)	Standard error	P-value
Model 1	0.029 (0.021, 0.036)	0.004	<0.001
Model 2	0.029 (0.022, 0.036)	0.004	<0.001
Model 3	0.007 (0.003, 0.011)	0.002	0.002
Model 4	0.005 (0.001, 0.010)	0.002	0.016

Model 1 was adjusted for age, sex. Model 2 was adjusted for age, sex, drink, smoke. Model 3 was adjusted for age, sex, drink, smoke, TG, Cr, SBP, FBG. Model 4 was adjusted for age, sex, drink, smoke, TG, Cr, SBP, FBG, BMI, ESR.

### Mediation analysis

**Figure 1:**
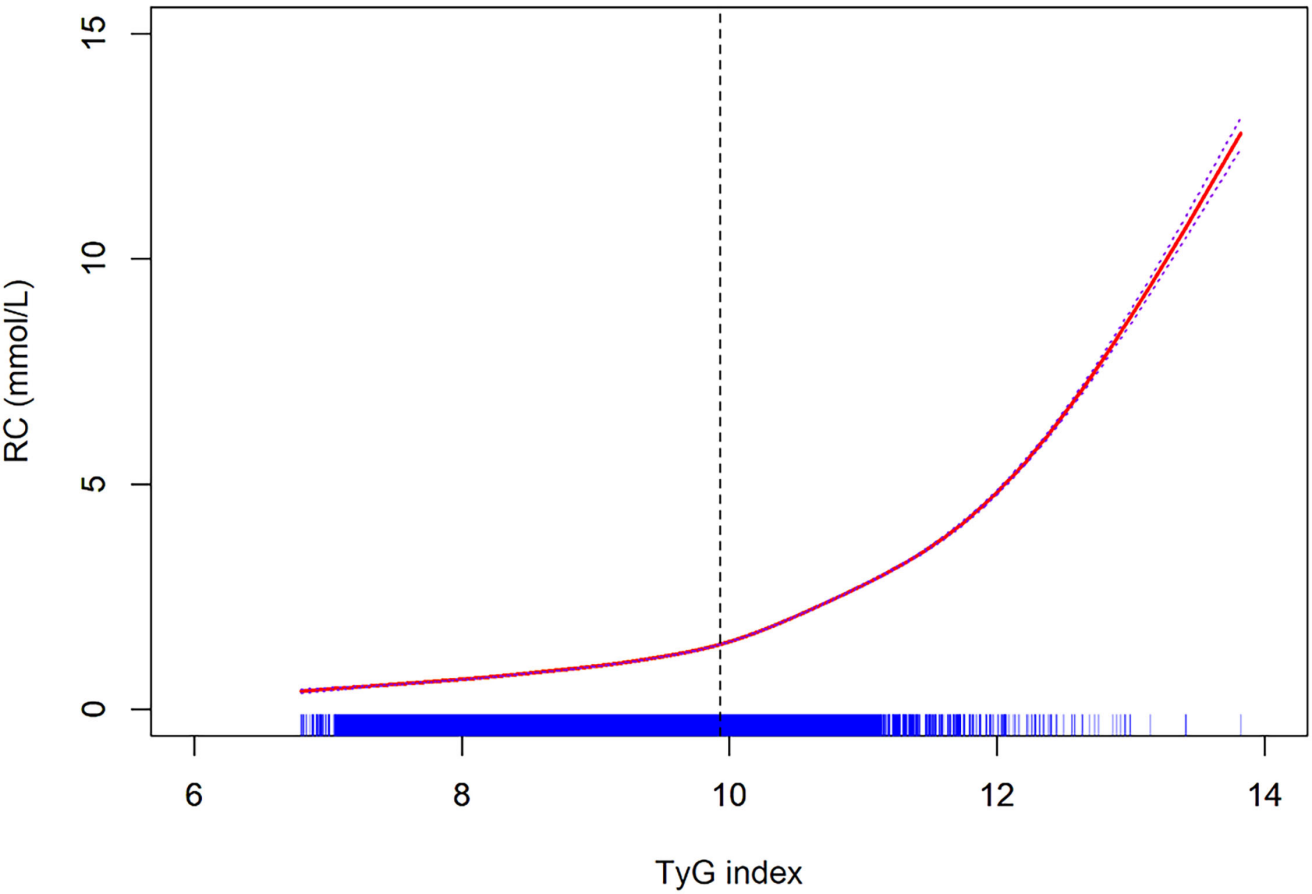
Non-linear relationship between TyG index and RC.

To further clarify the role of IR and inflammation in the association between *H. pylori* and RC, we performed a mediation analysis. After controlling for gender and age, findings revealed that the TyG index acts as a mediator in the relationship between *H. pylori* and RC, with a mediation proportion of 75.8% (95% CI = 63.1%-92.9%) (**Table 3**). Similarly, the ESR was identified as a mediator in this association, with a mediation proportion of 10.4% (95% CI = 7.4%-14.6%) (**Table 4**). Moreover, an increase in the TyG index was correlated with elevated RC levels; notably, when the TyG index surpassed 9.93, a significant increase in RC levels was observed (**Figure 1**).

**Table 3:** Mediation analysis of TyG index in the association between *H. pylori* and RC.

Mediation effect	Estimate	95% CI lower	95% CI upper	P-value
Total effect	0.029	0.021	0.036	<0.001
Direct effect	0.007	0.002	0.012	0.010
Indirect effect	0.022	0.017	0.026	<0.001
Proportion mediated	0.758	0.631	0.929	<0.001

**Table 4:** Mediation analysis of ESR in the association between *H. pylori* and RC.

Mediation effect	Estimate	95% CI lower	95% CI upper	P-value
Total effect	0.029	0.021	0.036	<0.001
Direct effect	0.026	0.018	0.033	<0.001
Indirect effect	0.003	0.002	0.004	<0.001
Proportion mediated	0.104	0.074	0.146	<0.001

### Subgroup analysis

**Figure 2:**
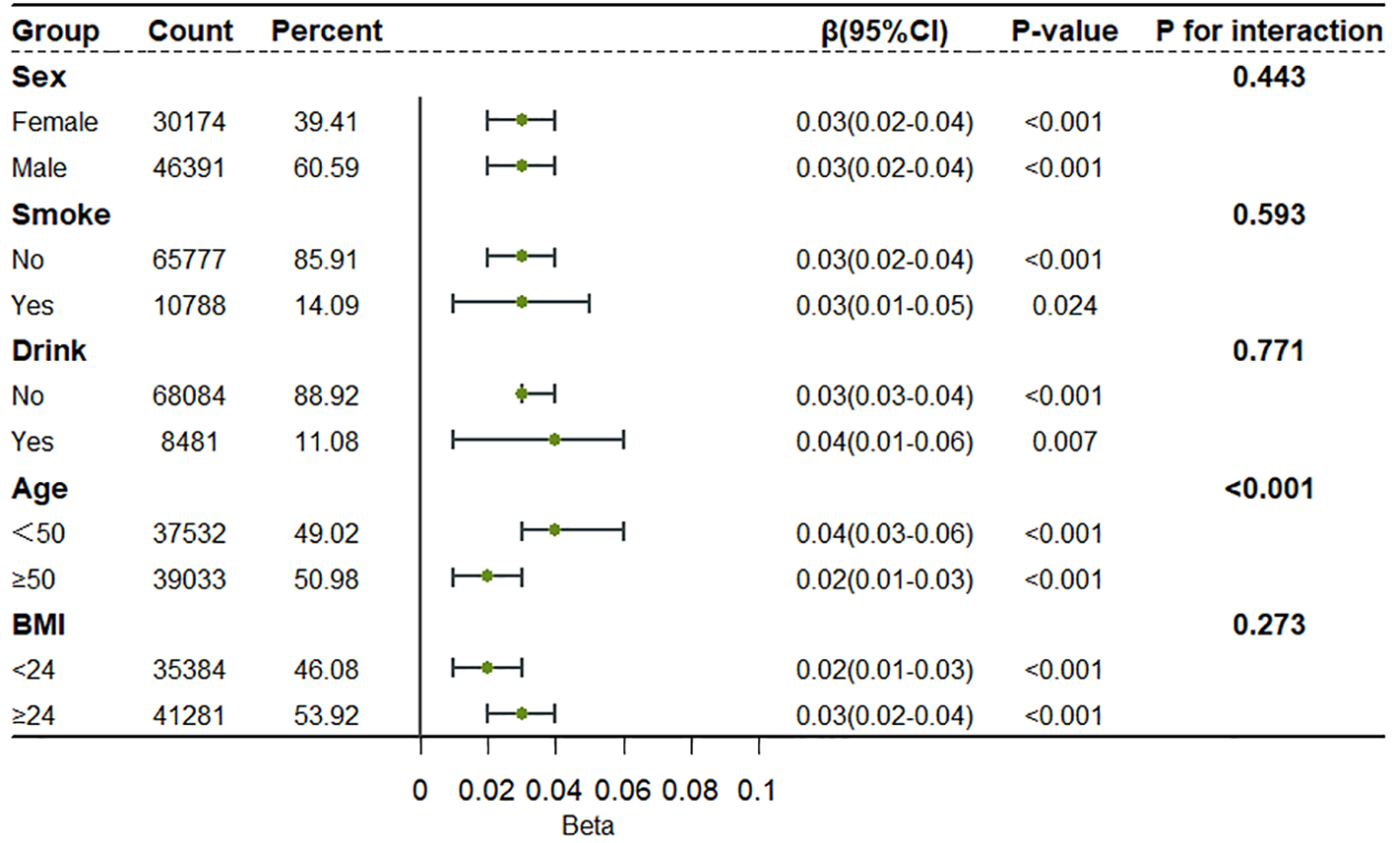
Subgroup analysis of the relationship between *H. pylori* infection and RC levels.

To better understand the association between *H. pylori* and RC across different populations, subgroup analyses were undertaken. Participants were stratified based on to sex, smoking, drinking, age, and BMI. The results indicated that *H. pylori* serves as a risk factor for RC in various populations; however, a significant interaction effect between age and *H. pylori* was identified, with a more pronounced impact in younger individuals (**Figure 2**).

### Longitudinal relationship between *H. pylori* and RC

**Figure 3:**
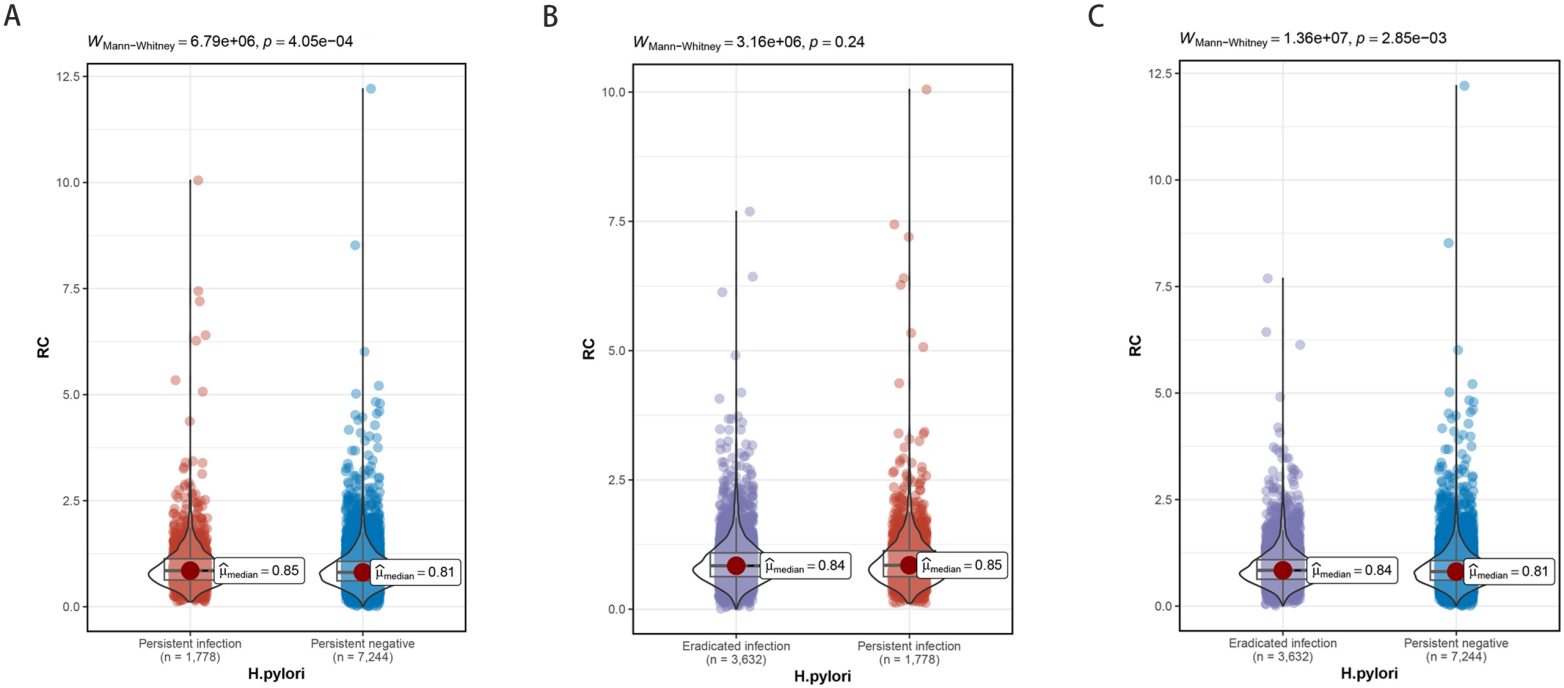
The effect of different *H. pylori* infection status on RC levels. **(A)** Persistent infection group compared with persistent negative group. **(B)** Eradicated infection group compared with persistent infection group. **(C)** Eradicated infection group compared with persistent negative group. Group differences were assessed using the Mann-Whitney tests, with test statistics and P values shown.

The study cohort was observed over a median follow-up duration of 3.2 years. The persistent infection group showed significantly higher RC levels compared to the persistent negative group (**Figure 3A**). Although the eradicated infection group exhibited lower RC levels than the persistent infection group, this difference was not statistically significant (**Figure 3B**). Nonetheless, RC levels in the eradicated infection group were elevated in comparison to the persistent negative group (**Figure 3C**).

## Discussion

Cardiovascular diseases contribute significantly to global mortality and disability, leading major economic burdens (Roth et al., 2020). Recently, RC has been recognized as a critical metabolic marker for evaluating cardiovascular residual risk, especially in individuals with multiple metabolic disorders or those at high cardiovascular risk, where its clinical assessment and intervention potential are significant (Huh et al., 2022; Wadström et al., 2023a). This study systematically examined the link between *H. pylori* infection and RC levels utilizing data from a large-scale health screening population for the first time. The results showed a significant association between *H. pylori* infection and higher RC levels, with this relationship holding statistical significance even after considering a range of potential confounders.

*H. pylori* is acknowledged as a persistent infectious agent, with its pathogenic mechanisms having been extensively investigated (Camilo et al., 2017; de Brito et al., 2019). It prompts the release of inflammatory substances like interleukin-6 and tumor necrosis factor-alpha, thereby activating a low-grade chronic inflammatory response (Cheng et al., 2017). This process may disrupt host immune homeostasis and metabolic pathways, potentially leading to dyslipidemia. The present study identified that people infected with *H. pylori* exhibit significantly elevated RC levels, while LDL and TC levels are reduced in these individuals. This finding may underscore the independent significance of RC as a non-traditional lipid marker in predicting cardiovascular residual risk. Mediation analysis findings showed that the TyG index is a crucial mediator linking *H. pylori* to RC levels, implying that IR could be the pivotal mechanism through which *H. pylori* impacts RC levels. Researches indicate that *H. pylori* can lead to a reduction in the phosphorylation of insulin signaling proteins, thereby hindering the activation of downstream proteins and inducing IR (Zhou et al., 2015; He et al., 2016). In IR states, the capacity of adipose tissue to respond to insulin and inhibit lipolysis is compromised, resulting in an increased release of free fatty acids (FFAs) (Avramoglu et al., 2006; Choi and Ginsberg, 2011). These FFAs are subsequently absorbed by the liver, where they are re-esterified into TG, thus promoting the synthesis and secretion of VLDL (Choi and Ginsberg, 2011). As the primary precursor of RC, an increase in VLDL provides a foundation for elevated RC levels. Furthermore, this study identified that the ESR mediates the relationship between *H. pylori* and RC levels, underscoring the role of systemic inflammation as a significant pathway in this association. Regarding lipoprotein metabolism, inflammation is posited to inhibit lipoprotein lipase activity, delay VLDL clearance, and enhance cholesteryl ester transfer protein activity, ultimately contributing to the accumulation of RC in TG-rich particles (Dallinga-Thie et al., 2010; Ginsberg et al., 2021; Yanai et al., 2022). Additionally, it has been proposed that *H. pylori*-induced intestinal dysbiosis may influence lipid metabolism through the gut-hepatic axis, a mechanism that warrants further investigation (Martin-Nuñez et al., 2021; Nabavi-Rad et al., 2022).

The subgroup analysis of this research further indicated that the relationship between *H. pylori* infection and RC levels was relatively consistent across various populations. However, it was notably more pronounced in younger individuals compared to older ones. This phenomenon may be attributed to the heightened sensitivity of metabolic systems and the increased activation of the immune system in younger individuals. Although some large observational studies have reported sex differences in the prevalence of *H. pylori* infection, their influence on downstream metabolic consequences remains inconclusive (Ibrahim et al., 2017). In the present study, the association between *H. pylori* infection and RC elevation was comparable in men and women, suggesting that metabolic pathways linking infection and lipid dysregulation may be largely sex-independent. Longitudinal analysis revealed that the elevation of RC levels was more significant among individuals with persistent *H. pylori* infection. Conversely, although RC levels exhibited a slight decrease in the group where the infection had been eradicated, they continued to show an upward trend compared to those with persistent negative status. This finding implies that the impact of *H. pylori* infection on RC may be delayed or partially irreversible. This observation aligns with previous reports indicating limited metabolic improvement following *H. pylori* eradication, underscoring the importance of early screening and intervention (Upala et al., 2017).

This research assessed the association between *H. pylori* infection and RC levels with a large population sample, utilizing mediation analysis to explore potential underlying mechanisms. Nonetheless, certain limitations persist. Firstly, despite controlling for various potential confounding factors, unmeasured residual confounders related to lifestyle and dietary patterns may still impact the results. Secondly, as this investigation is a single-center study, additional multi-center studies are required to enhance generalizability. Furthermore, the absence of typing data for *H. pylori* virulence factors, such as VacA and CagA, limits the ability to differentiate the effects of various strains on RC levels.

In summary, this study is the first to identify a significant positive association between *H. pylori* infection and RC levels within a large-scale population, and further revealed the potential mechanism of inflammation and IR in this association through mediation modeling. The above findings not only enrich the perspective of systemic metabolic effects of *H. pylori*, but also suggest that it may be an important modifier of residual cardiovascular risk. This provides a theoretical foundation for future investigations into *H. pylori* as a potential target for interventions in cardiovascular and metabolic diseases. Future studies should conduct prospective follow-up and randomized intervention trials in multi-center populations to assess the long-term effects of *H. pylori* eradication therapy on RC levels and downstream atherosclerosis risk, and to provide theoretical basis and evidence-based support for its incorporation into comprehensive cardiovascular disease management strategies.

## Conclusion

Our research identified an association between *H. pylori* infection and increased levels of RC, with IR and inflammation acting as mediating factors in this relationship.

## Data Availability

The raw data supporting the conclusions of this article will be made available by the authors, without undue reservation.

## Supplementary material

Supplementary Figure 1 Correlation of various risk factors.
