# Supplementary figures and images for: Relationship between Helicobacter pylori infection and remnant cholesterol: the mediating role of insulin resistance and inflammation
Source: Front Cell Infect Microbiol. 2025 Dec 19;15:1684556. doi: 10.3389/fcimb.2025.1684556 (PMC12757358; doi:10.3389/fcimb.2025.1684556)

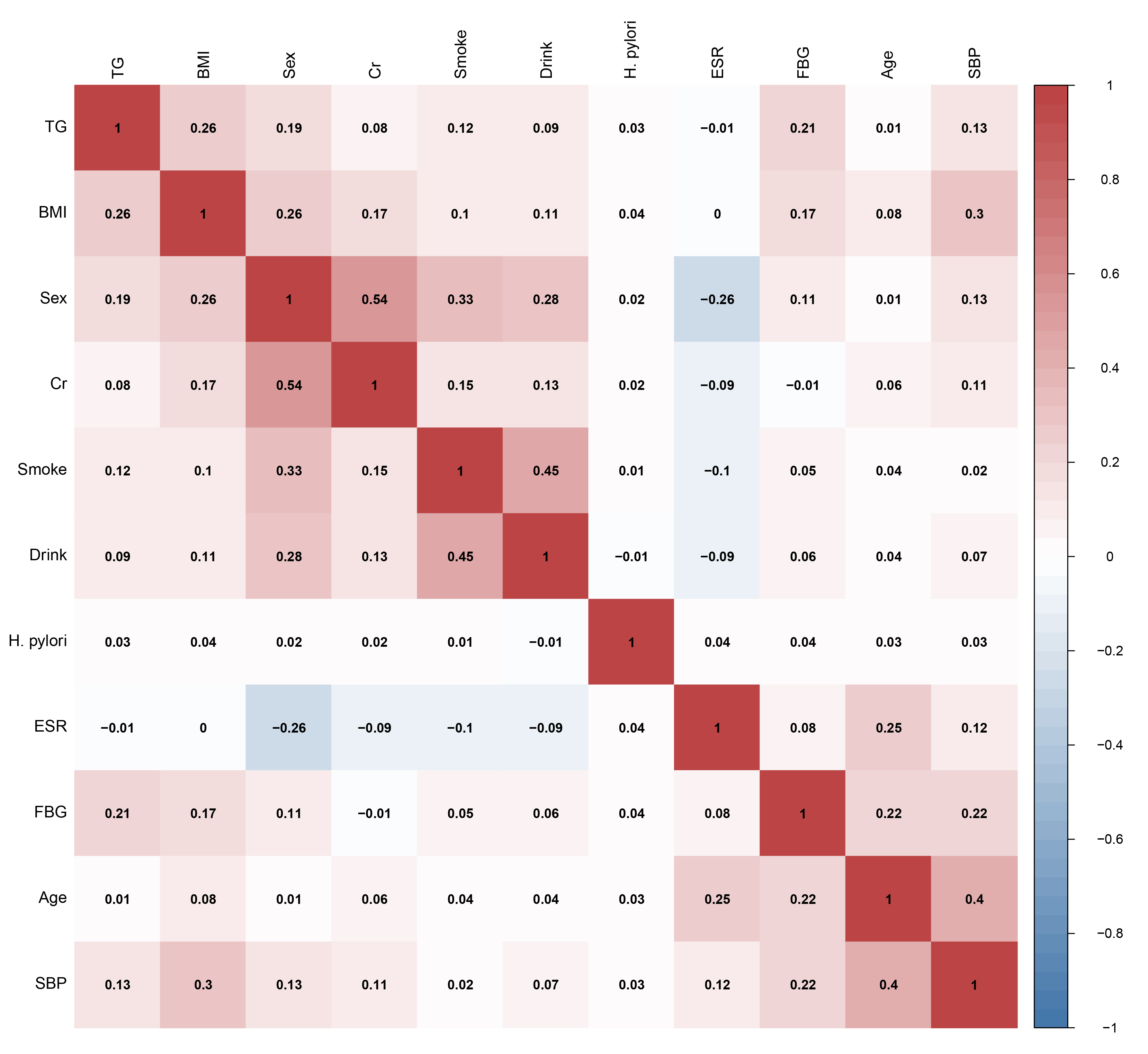

Supplement: Supplementary Figure 1 — Correlation of various risk factors. [file Image1.jpg]
